# Mean serum-level of common organic pollutants is predictive of behavioral severity in children with autism spectrum disorders

**DOI:** 10.1038/srep26185

**Published:** 2016-05-13

**Authors:** Andrew Boggess, Scott Faber, John Kern, H. M. Skip Kingston

**Affiliations:** 1 Department of Chemistry and Biochemistry, Duquesne University, Pittsburgh, PA, USA; 2 The Children’s Institute of Pittsburgh, Pittsburgh, PA, USA; 3 Department of Mathematics and Computer Science, Duquesne University, Pittsburgh, PA, USA.

## Abstract

Autism spectrum disorders (ASD), and their pathogenesis, are growing public health concerns. This study evaluated common organic pollutant serum-concentrations in children, as it related to behavioral severity determined by rating scales and the Autism Diagnostic Observation Schedule (ADOS). Thirty children, ages 2–9, with ASD and thirty controls matched by age, sex, and socioeconomic status were evaluated using direct blood serum sampling and ADOS. Pooling concentrations of all studied pollutants into a single variable yielded cohort-specific neurobehavioral relationships. Pooled serum-concentration correlated significantly with increasing behavioral severity on the ADOS in the ASD cohort (p = 0.011, r = 0.54), but not controls (p = 0.60, r = 0.11). Logistic regression significantly correlated mean pollutant serum-concentration with the probability of diagnosis of behaviorally severe autism, defined as ADOS >14, across all participants (odds ratio = 3.43 [95% confidence: 1.14–10.4], p = 0.0287). No specific analyte correlated with ADOS in either cohort. The ASD cohort displayed greater quantitative variance of analyte concentrations than controls (p = 0.006), suggesting a wide range of detoxification functioning in the ASD cohort. This study supports the hypothesis that environmental exposure to organic pollutants may play a significant role in the behavioral presentation of autism.

Emergent data have implicated environmental exposure, evidenced by serum-concentrations of specific environmental pollutants, as a correlative factor in the etiology of numerous non-communicable diseases, such as autism spectrum disorder (ASD), heart disease, diabetes, and lupus^1^,^2^,^3^,^4^. Exposure to heavy metals and organic pollutants, collectively termed xenobiotics, has been linked with neurodegenerative diseases, including Parkinson’s and Alzheimer’s diseases, suggesting the central nervous system is specifically susceptible^5^,^6^.

Autism spectrum disorders (ASD) are characterized behaviorally by impaired social communication and interaction, and repetitive behaviors^7^. Recent studies have suggested the incidence of ASD is increasing^8^, but scholarly opinion remains divided on the mechanism governing the increase^9^. While studies have estimated that at least half of the etiology of ASD may be attributable to genetic factors^10^, it has been suggested that ASD development and maintenance may be associated with exposure to environmental triggers^11^,^12^. Studies have correlated maternal exposure to environmental air pollutants and proximity to coal-fired power plants, pesticide-rich agricultural fields, known toxic-chemical sites, and traffic-related air pollution with increased rates of ASD^13^,^14^,^15^,^16^,^17^,^18^. Noting that environmental exposures typically occur in combination, not isolation, researchers have recommended that greater focus be placed on examining the combinative physiological effects of organic pollutants^19^.

Researchers have proposed that a portion of the pathophysiology of ASD may relate to interactions between physiology and the environment in genetically predisposed or otherwise susceptible individuals. A fundamental expectation in this hypothesis is the observation of differential physiological response to similar environments by susceptible individuals compared with typical controls. A number of pathways have been proposed to explain this physiological/environmental interaction hypothesis, focusing primarily on impaired methylation and detoxification that may lead to glutathione-redox imbalance and central nervous system excitotoxicity. Studies have discovered each of these features in individuals with ASD^20^,^21^,^22^,^23^,^24^.

Methionine (Met) is an essential amino acid that acts as a methyl group donor to facilitate cellular methylation to, in part, protect, stabilize, and epigenetically activate or deactivate DNA. Environmentally-sourced xenobiotics have been shown to inhibit the activity of methionine synthase (MS)^25^ the enzyme responsible for converting homocysteine to Met. Exposure to some xenobiotics may, therefore, reduce cellular methylation capabilities in numerous systems that require Met. One important methylation pathway is dopamine-stimulated phospholipid methylation (PLM), which utilizes a D4 subtype dopamine receptor that is critical for synchronization of brain activity during attention^26^,^27^. This suggests that xenobiotic impairment of MS could limit dopamine-stimulated PLM, leading to impairments in neuronal synchronization and attention, key features found in children with ASD^20^,^28^.

Detoxification is the process by which xenobiotics are removed from the body and is dependent on Met and MS. The primary antioxidants, Met and glutathione (GSH), are sulfur-containing antioxidants that act as a primary pathway for removal of xenobiotics from tissues, regardless of chemical identity. Total stress on detoxification pathways may be more dependent on overall xenobiotic insult than on specific xenobiotics^20^. Impaired MS activity may limit the detoxification capacity of the body by reducing the functioning of sulfur-containing antioxidant pathways.

Met displays moderate antioxidant capacity and acts as a biochemical precursor to the primary bodily antioxidant, GSH. Balance between reduced glutathione (GSH) and oxidized glutathione (GSSG) has a broad impact on overall cellular health. Met protects GSH/GSSG redox balance by preventing GSH depletion, as GSH is synthesized in a reaction involving Met. An imbalance in GSH/GSSG and an increase in reactive species in the body is called oxidative stress. Oxidative stress can allow xenobiotics, specifically organochloride pesticides and polychlorinated biphenyls, to disrupt the neurotransmitters glutamate and gamma-aminobutyric acid (GABA) and lead to central nervous system excitotoxicity, which can exacerbate symptoms of ASDs^21^. This suggests that impaired MS activity could create competition for available GSH in the GSH/GSSG redox-cycle^22^, resulting in increased oxidative stress and greater potential for central nervous system excitotoxicity.

The proposed physiological/environmental interaction hypothesis suggests that environmental insult may impair MS activity and affect multiple systems simultaneously by decreasing methylation capabilities, inducing or exacerbating detoxification dysfunction, and limiting dopamine-stimulated PLM, ultimately contributing to neurological symptoms associated with ASD.

Some studies have implicated specific xenobiotics as correlative factors in ASD etiology. The purpose of this case-control study was to evaluate the relationship between organic pollutants and behavioral severity in children with ASD and matched controls. With this objective, direct chemical measurements of non-metabolized organic pollutants and behavioral rating scales/observations were conducted in children diagnosed with ASD (n = 30) and controls (n = 30) matched by age, sex, and socio-economic status in Western Pennsylvania to investigate the relationship between organic pollutants and neurological symptoms associated with ASD.

## Results

A complete portfolio of individuals included in this study can be found in Table 1 and displays pairing parameters including age, sex, and ADOS score. The analytical method was validated using blank-subtracted serum spiked with certified concentrations of each studied xenobiotic prior to this study to assess accuracy, precision, and limits of detection and quantitation. All analytes demonstrated a mean accuracy of <8% error and mean precision of <10% RSD.

### Qualitative Assessment of Exposure

**Figure 1:**
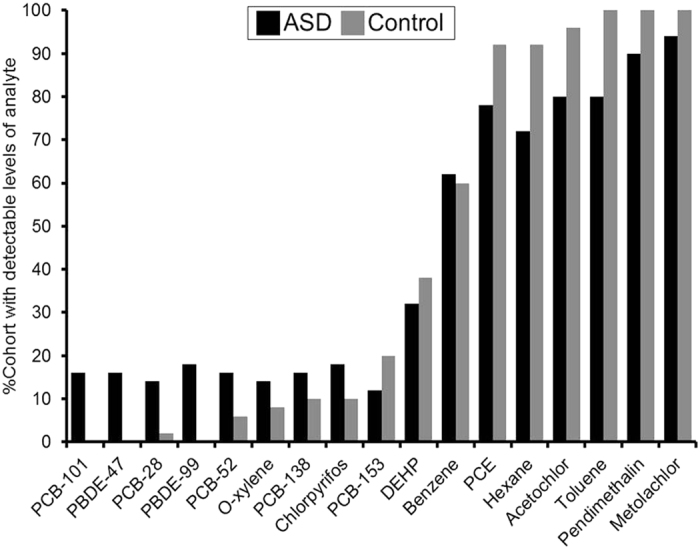
Percentage of the total population displaying detectable amounts of each compound, showing portion of compound detected in each cohort.

Detection results can be found in Fig. 1, showing percentages of each cohort with detectable amounts of specified xenobiotic in their serum. Perchloroethylene (PCE), n-hexane, benzene, and toluene, were detected in >80% of all children tested. Acetochlor, metolachlor, and pendimethalin were detected in >90% of the children in the study. PCBs 153, 138, 52, 28, and 101 were detected in 50%, 17%, 14%, 12%, and 9% of all children in the study, respectively.

PCB-101, PBDE-47, and PBDE-99 were only detectable in the ASD cohort. PCB 138 was detected in 1.5-times more individuals in the ASD cohort than controls. The pesticides acetochlor, pendimethalin, and metolachlor were detected in a greater number of controls than the ASD cohort; 20%, 8%, and 4% greater, respectively. Overall, the cohort with ASD contained a significantly greater number of children with detectable levels of PCBs and PBDEs (p = 0.0359, p = 0.00730). Detection rates of pesticides, volatile organic compounds, and solvents did not differ to a statistically significant degree between the two cohorts.

### Quantitative Cohort Comparisons

Full quantitative results and statistical tests for each xenobiotic by cohort can be found in Table 2. The pesticide metolachlor was quantified at a higher mean concentration in the control cohort than the cohort with ASD (p = 0.021). The pesticide pendimethalin trended toward higher concentrations in the control cohort, outside of statistical significance (p = 0.118). No other significant concentration differences were discovered between the two cohorts for any specific xenobiotic. No statistically significant correlations were discovered between or among individual xenobiotics or rating scales. The results for analytes PCB-28, PCB-52, PCB-101, PCB-138, and PCB-152, though qualitatively detectable in many participants (see: Qualitative Assessment of Exposure), were not significant enough to yield quantifiable data in any participant.

Comparing the pooled mean of all compounds from the ASD cohort to the pooled mean for all compounds in the control cohort yielded no significant difference (p = 0.846). O-xylene, PBDE-47, and PBDE-99 were quantifiable in <5 individuals in each cohort and were not included in data pooling statistics to eliminate bias toward any specific compound, leaving nine xenobiotics with which to compare quantitative variance.

### Quantitative Variance

Detailed variance and relative standard deviation (%RSD) data can be found in Table 3. Overall, the cohort with ASD demonstrated a greater range of concentrations for most of the xenobiotics studied compared with controls. The cohort with ASD displayed significantly greater variance in the pooled mean of all compounds compared with controls (p = 0.006) and greater %RSD for seven of the nine xenobiotics included in the variance analysis.

### Mean Xenobiotic Body-Burden and Behavioral Severity

All individuals in the ASD cohort scored 7 or higher on total ADOS, meeting the cutoff for ASD diagnosis. All individuals in the control cohort achieved a score of 6 or less. No significant correlations were observed between specific xenobiotic concentrations, MXB, or rating scale performance with age, ethnicity, sex, or socio-economic status, indicating matched-pairing was statistically irrelevant.

**Figure 2:**
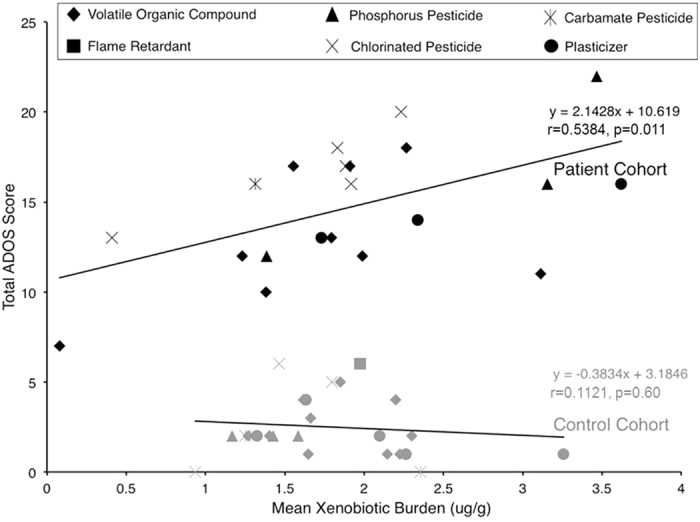
Individual total ADOS performance as a function of mean xenobiotic burden in serum for ASD cohort and controls, showing the compound class with the greatest relative contribution to mean.

The exclusion criteria for MXB eliminated nine from the ASD cohort and six controls from this analysis due to insufficient data. Total ADOS performance as a function of MXB for each individual produced two disparate trends, demonstrated in Fig. 2. The ASD cohort (n = 21) exhibited a modest positive linear correlation between increasing MXB and total ADOS performance (r = 0.5384, p = 0.011), while the control cohort (n = 24) demonstrated no linear trend or correlation with MXB (r = 0.1121, p = 0.60). MXB significantly correlated with the two ADOS sub-domains: communication (p = 0.020) and social (p = 0.050) in the cohort with ASD, but not controls (p = 0.80, p = 0.34, respectively).

No parent-administered questionnaires or rating scales produced significant correlations with MXB in either cohort. As seen in Table 4, Hexane, PCE, DEHP, and chlorpyrifos exhibited the greatest influence on MXB, as shown by the strength and direction of correlation between MXB and these four xenobiotics. No specific xenobiotics correlated significantly with total ADOS performance in either the ASD or control cohorts.

Mean MXB of individuals with total ADOS >14 was significantly greater than the control cohort (p = 0.029) and the rest of the ADOS ≤14 study-population (n = 34, p = 0.018). Within the ASD cohort, mean MXB of individuals with total ADOS ≤14 (n = 10) trended lower than those with total ADOS >14 (n = 11, p = 0.054). Mean MXB of the ASD cohort did not differ significantly from mean MXB of the controls (p = 0.491). A logistic regression was performed with four rare-event adjustments. Across all eligible participants (n = 45), MXB was found to be a predictor variable for probability of obtaining a total ADOS >14 (O.R.  = 3.43, 95% confidence: 1.14–10.4) with a statistically significant overall fit (p = 0.028, coefficient: 1.23).

## Discussion

Researchers have suggested that children with ASD may have dysfunctional cellular-level pathways necessary for removal of xenobiotics from the body, though concerns have been expressed over the design and methodologies of some studies^29^. The work discussed here addressed many of these concerns by measuring environmentally relevant serum-concentrations and using peer-matched controls to investigate relationships between and among organic pollutants and autism rating scales/observations.

This study supports the hypothesis that a portion of the pathophysiology leading to autism may be attributable to exposure to organic chemical pollutants. Evidence of differing efficacies of detoxification and methylation systems between the two cohorts could be suggested by three separate findings: quantitative and qualitative differences in specific organic pollutants, significantly different quantitative variance, and significantly altered levels of methylcobalamin (MeCbl) in the two cohorts (based on previously published research)^30^. Differential neurobehavioral response to MXB between the two cohorts further suggests a relationship between the pathophysiology of autism and general exposure to organic chemical pollutants.

All participants in this study lived in Western Pennsylvania and may have been exposed to similar outdoor environments, though indoor environments and diet were not specifically controlled by this study. The cohorts exhibited quantitative and qualitative differences in specific xenobiotics. The control cohort displayed a significantly greater serum-concentration of metolachlor, a common pesticide in Western Pennsylvania. Qualitatively, a greater percentage of the ASD cohort had detectable PCBs. Previous studies have noted that elevated PCBs can increase intraneuronal calcium and alter calcium signaling via changes in the ryanodine receptor, which can upset the balance between glutamate and GABA neurotransmission, creating central nervous system excitotoxicity, a feature commonly found in children with autism^19^,^31^.

Researchers associated with the current study had previously found significantly greater levels of methylmalonic acid (MMA) in the cohort with ASD compared with controls in a concurrent study utilizing identical participating children^30^. Increased MMA indicates deficiency in MeCbl, a form of vitamin-B_12_, contributing to impaired MS function^31^. Impaired MS function could disrupt multiple pathways associated with both detoxification and methylation in the children with ASD, including dopamine-stimulated PLM necessary for neuronal synchronization and normal functioning of the central nervous system^27^.

With the exception of metolachlor, no other concentration differences were observed between the cohorts for specific xenobiotics. However, the ASD cohort displayed significantly greater variance around the mean of individual organic pollutants, as well as the pooled mean, compared with controls. This difference in variance suggests the children with ASD may have had greater variability in detoxification efficacy compared with controls^32^.

Differing neurobehavioral trends in relation to MXB were observed in the two cohorts. The strong correlation between MXB and all three ADOS markers (total, social, and communication) in the ASD cohort, but not controls, is a fundamental expectation from the hypothesis of genetic predisposition for susceptibility to environmental triggers.

A total of 9 children from the ASD cohort and 6 from controls were excluded from the MXB vs. ADOS and logistic regression calculations based upon the discussed MXB exclusion criteria to prevent introduction of bias. While these exclusion criteria were based upon cited and accepted statistical methods, it must be noted that these exclusions themselves could have introduced bias, given the significant percentage of the total population meeting the criteria. The limited sample size and further reduction by exclusion is a primary limitation of this study. Future work would benefit from increased sample population to allow necessary exclusion while reducing potential bias.

## Methods

The study was approved by the Institutional Review Board (IRB) of Duquesne University (Pittsburgh, PA, USA) (IRB #10–36) and was conducted at The Children’s Institute of Pittsburgh. All aspects and methods of this study were carried out in accordance with with the approved guidelines. All analysts involved with sample processing and chemical quantitation remained blind to the identification and ASD-diagnosis status of all individuals throughout the course of the study and data analysis.

### Subject enrollment

Informed consent was attained from all participants in this study in all cases. The parents of thirty children aged 2–9 with autism or pervasive developmental disorder-not otherwise specified (PDD-NOS), who were evaluated at the Neurodevelopmental Service of The Children’s Institute, reviewed the inclusion and exclusion criteria with a research coordinator. The policies outlined in the Common Rule of Responsible Conduct of Research in Humans and the Declaration of Helsinki were followed. Further, parent consent was attained and, where possible, child assent was gained. A psychologist with research level certification in performance of the Autism Diagnostic Observation Schedule (ADOS) met with each child and a diagnosis of autism or PDD-NOS allowed participation in the study. A total of 31 children with autism were evaluated by the clinician, with one family of a child from the ASD cohort choosing to leave the study prior to the blood draw and biochemical assessment. Thirty children in the control group were recruited by a poster and matched by age, sex, and socioeconomic status with the thirty children who met criteria for PDD-NOS or autism.

Following consent and child assent, control children underwent a history and physical examination to rule out genetic, developmental, neuropsychiatric disorders, and an immediate family member with ASD. Control children then underwent identical ADOS evaluation, which confirmed the absence of autism.

### Behavioral Rating Scales and Observations

The ADOS is a widely accepted standard measure of deficits associated with ASDs^33^. Nomenclature at the time of the study supported a total score of 7 indicating the presence of ASD^33^. This study utilized the ADOS-suggested categorizations, with a total score of 7 indicating the presence of ASD and a total score of 14 differentiating individuals into the most behaviorally severe subset for data processing purposes.

Behavioral rating scales were filled out by a parent or guardian. The rating scales included the Social Communication Questionnaire (SCQ), Aberrant Behavior Checklist (ABC), Gilliam Autism Rating Scale (GARS), Autism Treatment Evaluation Checklist (ATEC), PDD Behavior Inventory (PDDBI), and Childhood Autism Rating Scale (CARS). For analytical comparisons, three ADOS observational results, including the communication and social domain values, as well as the total ADOS score (a sum of the communication and social domain scores) were determined.

### Participant Blood Draw

Approximately 30 mL of blood was drawn at each child’s home and was processed for storage within two hours. The blood was centrifuged and aliquots were separated into serum and red cells, before storage at −80 °C. All blood processing and analysis was performed in a laboratory certified as an International Organization for Standardization Class-5 cleanroom. The serum was processed from whole blood that was drawn using tubes specifically certified free of volatile organic compounds.

### Analyte Selection

Previous studies guided the selection of organic xenobiotics associated with the etiology of ASD^34^,^35^. The selected analytes represented a wide range of volatility, chemical function, and hydrophobicity. Three volatile organic compounds (VOC), benzene, toluene, and *o-*xylene; one alkane, hexane; five polychlorinated biphenyls (PCB), IUPAC congeners 28, 52, 101, 138, and 153; two polybrominated diphenylethers (PBDE), IUPAC congeners 47 and 99; two organochlorine pesticides, metolachlor and acetochlor; one dinitroanaline pesticide, pendimethalin; one organophosphate pesticide, chlorpyrifos; one phthalate, bis (2-ethylhexyl) phthalate (DEHP); and the chlorocarbon perchloroethylene were chosen for this research.

### Instrumental and Quantitative Methods

All serum samples were assigned a randomized identification number and all analysts were blinded to the identity of samples. Serum samples were analyzed by previously peer-reviewed methods^36^. Chromatography and mass spectrometry were conducted using an Gas Chromatograph –Mass Spectrometer system. Analytes were introduced into the GC-MS thermally using a thermal desorption unit (TDU) (GERSTEL GmbH & Co., Mülheim, Germany). Method blanks were run between replicates and instrument blanks between each new sample. Quantitation was accomplished using the standard method of isotope dilution-mass spectrometry (IDMS), defined in EPA Method 6800.

Teflon-lined screw caps were fixed to the vials. Vials were stirred at 1500 rpm for 60 minutes. The stir-bars were removed from the sample with tweezers and thoroughly rinsed with ultrapure water and dried with a lint-free tissue and deposited into a glass thermal desorption tube. This single-bar method was developed for use with all analytes in this study. However, quantification of the VOCs was completed before development of the single-bar method. Therefore, benzene, toluene, and o-xylene were extracted by the traditional dual stir-bar method for both immersive and headspace extraction.

The GC inlet was set to programmed-temperature vaporization (PTV) with a chilled injector system (CIS-6) (GERSTEL) inlet packed with Tenax TA (Buchem B.V., Apeldoorn, The Netherlands). A robotic multipurpose sampling system was used for sample handling and injecting. A final desorption temperature of 280 °C was used in the TDU before cryofocusing at −50 °C in the PTV system with liquid nitrogen. Finally, the CIS system was heated at 720 °C/minute to 280 °C to transfer the analytes for analysis. Chromatography was performed on an HP-5 MS column (30 m × 0.25 mm I.D. 0.25 μm film thickness, 5%-phenyl polydimethylsiloxane) at a 1.0 mL/minute carrier gas flow rate. The GC oven was heated from 45 °C to 280 °C at 12 °C per minute, and held for 15 minutes.

### Statistics and Validation

All participants were analyzed with n = 5 replicate subsamples. Method blanks were analyzed between each sample and analytical blanks were analyzed between subsamples. Means and standard deviations were calculated using n = 5 replicates. The lower limit of quantitation (LOQ) was calculated as ten times the standard deviation of repetitive measurements of the blank. All statistical comparisons were performed at the 95% confidence interval (CI). Variance (σ^2^) was assessed as the square of standard deviation. Accuracy was evaluated by determining percent error from a certified value. For cohort comparisons, a standard p-value cutoff was set as 0.05. All p-values are reported as two-tailed. Validation was performed by spiking certified standards for each compound into blank-subtracted blood serum and analyzing by the above method (n = 5). Experimental concentrations were statistically compared with certified values (n = 5).

### Mean Xenobiotic Body-Burden and Exclusion Criteria

Concentrations of all xenobiotics quantified in an individual were pooled into one variable, termed mean xenobiotic body-burden (MXB), for each individual. The MXB value of an individual averaged only those xenobiotics that were quantified above the compound-specific LOQ. Following peer-reviewed methods for handling missing variables in pooled data, individuals possessing quantifiable concentrations in <50% of the studied xenobiotics were excluded from the MXB comparative analyses^37^.

### Linear and Logistic Regressions

Linear regression was performed on the separate cohorts to determine the relationship between total ADOS score and MXB. Logistic regression was performed and all probabilities were reported in decimal form. A peer-reviewed method for adjusting logistic regression for rare-event data was used^38^. In this way, adjusted regressions were produced assuming 0.5%, 1%, 2%, or 3% national prevalence of the most severe autism diagnosis.

## Conclusion

The results from this study were unique in finding the mean burden of organic pollutant concentrations in an individual to significantly correlate with behavioral severity in children with ASD, but not controls. The mean xenobiotic burden also significantly correlated with increased risk of the most behaviorally severe ASD diagnosis across both cohorts with an odds ratio of 3.4. With the exception of metolachlor, results indicated the cohorts had statistically similar concentrations of individual organic pollutants studied; however, the ASD cohort demonstrated a significantly greater range of concentrations. Studies that relate copy number variant data from oligoarrays, whole exome sequencing, and whole genomic sequencing to efficacy of methylation and detoxification processes and behavioral phenotype are reasonable next steps in the evaluation of the hypothesis that environmental triggers contribute to the etiology and maintenance of autism spectrum disorders.

## Additional Information

**How to cite this article**: Boggess, A. *et al*. Mean serum-level of common organic pollutants is predictive of behavioral severity in children with autism spectrum disorders. *Sci. Rep.* **6**, 26185; doi: 10.1038/srep26185 (2016).

## Figures and Tables

**Table 1:** Matched individuals included in this study and parameters included in pairing.

**Case Cohort**				**Control Cohort**			
**ID**	**Age**	**ADOS**	**Sex**	**ID**	**Age**	**ADOS**	**Sex**
7	2	18	M	17	2	1	M
20	2	12	F	36	2	2	F
30	2	18	M	52	2	1	M
5	3	11	M	19	3	1	M
10	3	20	M	22	3	2	M
13	3	16	M	31	3	0	M
21	3	10	M	41	3	2	M
43	4	10	M	49	4	3	M
3	5	21	M	28	5	6	M
9	5	12	M	29	5	5	M
12	5	17	M	42	5	6	M
24	5	17	M	45	5	4	M
26	5	17	M	46	5	2	M
32	5	13	M	51	5	5	M
47	5	16	M	53	5	5	M
2	6	10	M	37	6	6	M
15	6	13	M	40	6	2	M
39	6	16	F	48	6	2	F
25	6	13	M	54	6	4	M
34	6	12	M	58	6	5	M
38	6	14	M	60	6	2	M
44	6	17	M	61	6	0	M
1	7	15	M	14	7	1	M
16	7	16	M	35	7	2	M
11	8	15	M	18	8	4	M
6	8	22	F	59	8	2	F
8	9	10	F	23	9	1	F
4	9	7	M	55	9	1	M
33	9	13	M	56	9	2	M
50	9	17	M	57	9	0	M
**Mean**	5.5	14.6			5.5	2.63	

**Table 2:** Full quantitative results by cohort for all xenobiotics included in the study.

	**ASD Cohort (μg/g)**		**Control Cohort (μg/g)**		p-value*
	**n**	**Mean (95% CI)**	**n**	**Mean (95% CI)**	
Benzene	4	0.263 (±0.132)	6	0.291 (±0.0870)	0.652
Toluene	26	0.143 (±0.100)	28	3.32 × 10^−2^ (±1.38)	0.123
o-Xylene	3	1.35 × 10^−3^ (±1.24)	0	–	–
Hexane	24	11.7 (±2.29)	29	9.44 (±0.810)	0.684
PCE	24	6.38 × 10^−2^ (±1.75)	27	5.64 × 10^−2^ (±2.50)	0.614
PBDE-47	1	0.00208 (N/A)	4	2.18 × 10^−3^ (±1.10)	–
PBDE-99	4	7.19 × 10^−3^ (±5.00)	3	1.41 × 10^−2^ (±0.890)	0.0987
Chlorpyrifos	10	2.27 × 10^−4^ (±1.11)	12	1.75 × 10^−4^ (±0.301)	0.891
Pendimethalin	28	1.07 × 10^−2^ (±1.08)	29	2.01 × 10^−2^ (±5.35)	0.118
Metolachlor	28	5.18 × 10^−3^ (±2.83)	30	1.20 × 10^−2^ (±0.514)	0.021
Acetochlor	25	0.182 (±0.0180)	30	0.160 (±0.0260)	0.695
DEHP	19	3.95 (±1.74)	19	3.29 (±1.05)	0.697
Pooled Mean	9	1.36 (±2.95)	9	1.21 (±2.39)	0.846

-Not enough detections for calculation.

^*^Comparing concentration from ASD cohort to control cohort.

**Table 3:** Cohort-specific differences in quantitative variance and relative standard deviation for each xenobiotic and pooled mean by cohort.

	**ASD Cohort**		**Control Cohort**	
	**Variance (σ**^**2**^)	**%RSD**	**Variance (σ**^**2**^)	**%RSD**
Benzene	0.0089	35.9	0.0075	29.7
Toluene	0.0849	172	0.00249	107
Xylene	5.94 × 10^−5^	57.0	–	–
Hexane	29.4	46.3	3.94	21.0
PCE	0.00171	64.9	0.00401	112
PBDE47	–	–	2.29 × 10^−7^	33.3
PBDE99	1.30 × 10^−5^	50.1	3.09 × 10^−5^	39.4
Chlorpyrifos	2.46 × 10^−8^	69.2	2.21 × 10^−9^	26.9
Pendimethalin	0.00079	263	0.0199	701
Metolachlor	5.26 × 10^−5^	140	0.000204	119
Acetachlor	0.00178	23.2	0.00542	46.0
DEHP	11.2	84.8	4.73	66.0
Pooled Mean^*^	4.07	288	0.871	262

*Variances statistically different between cohorts (p = 0.006).

-Not enough data to perform calculation.

**Table 4:** Strength and direction of correlations between specific xenobiotics with MXB and total ADOS for all analytes used in MXB calculation.

	**ASD Cohort**					**Control Cohort**			
	**Correlation with MXB**		**Correlation with total ADOS**			**Correlation with MXB**		**Correlation with total ADOS**	
	**r-value**	**p-value**	**r-value**	**p-value**		**r-value**	**p-value**	**r-value**	**p-value**
Hexane	0.77	<0.001	0.09	0.69	Hexane	0.57	0.003	−0.19	0.32
PCE	0.55	0.008	−0.11	0.61	PCE	0.40	0.053	−0.21	0.31
DEHP	0.51	0.02	0.023	0.93	DEHP	0.43	0.030	−0.060	0.83
Chlorpyrifos	0.46	0.032	0.29	0.44	Chlorpyrifos	−0.42	0.036	−0.34	0.27
Metolachlor	−0.31	0.16	0.041	0.84	Metolachlor	−0.020	0.90	−0.012	0.94
Pendimethalin	−0.16	0.48	0.15	0.43	Pendimethalin	0.12	0.56	−0.27	0.15
Toluene	−0.04	0.85	0.16	0.54	Toluene	0.13	0.53	−0.18	0.49
Acetachlor	0.088	0.70	0.012	0.95	Acetachlor	0.30	0.14	−0.18	0.34
Benzene	−0.089	0.64	−0.20	0.29	Benzene	−0.33	0.075	−0.23	0.22
